# Silent circulation of dengue virus in *Aedes albopictus* (Diptera: Culicidae) resulting from natural vertical transmission

**DOI:** 10.1038/s41598-020-60870-1

**Published:** 2020-03-02

**Authors:** Victor Henrique Ferreira-de-Lima, Pâmela dos Santos Andrade, Luciano Matsumiya Thomazelli, Mauro Toledo Marrelli, Paulo Roberto Urbinatti, Rosa Maria Marques de Sá Almeida, Tamara Nunes Lima-Camara

**Affiliations:** 1 Institute of Tropical Medicine, University of São Paulo. Av. Dr. Enéas de Carvalho Aguiar, 470 - Jardim América, São Paulo, SP 05403-000 Brazil; 2 Department of Epidemiology, School of Public Health, University of São Paulo. Av. Dr. Arnaldo, 715 - Cerqueira César, São Paulo, SP 01246-904 Brazil; 3 Laboratory of Clinical and Molecular Virology (LVCM), Institute of Biomedical Sciences II, University of São Paulo. Av. Prof. Lineu Prestes, 1374 - Butantã, São Paulo, SP 05508-900 Brazil

**Keywords:** Invasive species, Urban ecology

## Abstract

Vertical transmission in *Aedes aegypti* and *Aedes albopictus* is considered a maintenance mechanism for dengue virus (DENV) during unfavorable conditions and may be implicated in dengue outbreaks. Since DENV infection dynamics vary among wild-type viruses and vector populations, vertical transmission rates can also vary between regions. However, even though São Paulo is the most populous city in the Americas and has experienced major dengue epidemics, natural vertical transmission had never been detected in this area before. Here we confirm and describe for the first time natural vertical transmission of DENV-3 in two pools of male *Ae. albopictus* from the city of São Paulo. The detection of DENV-3 in years when no human autochthonous cases of this serotype were recorded suggests that silent circulation of DENV-3 is occurring and indicates that green areas may be maintaining serotypes that are not circulating in the human population, possibly by a vertical transmission mechanism.

## Introduction

Dengue is considered the most serious re-emergent viral disease transmitted by arthropods, with approximately 390 million infections worldwide each year, 7% of which occur in Brazil^1,2^. The etiological agent of the disease is an arbovirus of the genus *Flavivirus* and family Flaviviridae with four antigenically distinct serotypes (DENV-1, DENV-2, DENV-3 and DENV-4)^1,3,4^ which is mainly transmitted by the mosquito *Aedes aegypti*^5^ although *Aedes albopictus* plays an important role in transmission of this virus in numerous countries around the world, especially in Southeast Asia^5,6^.

In the Americas, the dengue cycle is essentially urban, having as fundamental elements of its transmission dynamics the etiological agent (DENV), the anthropophilic diurnal mosquito *Ae. aegypti* and the vertebrate host^5^ (humans). *Aedes albopictus* is considered an exophilic mosquito with eclectic feeding behavior^7,8^, but some degree of anthropophily has recently been shown for this species^9^. Because of its vector competence under laboratory conditions, *Ae. albopictus* is considered a potential vector of DENV^5,10^. The species has already been found naturally infected with DENV in a pineapple plantation in Costa Rica^11^, but this evidence is not sufficient for it to be considered an actual vector.

The most common way mosquito females become infected with an arbovirus is by blood feeding on a viremic host, which is known as horizontal transmission^12^. Another route of infection is transmission from the parents to part of the offspring, which is known as vertical transmission^13,14^. While in endemic areas the ability of DENV to persist in the environment during periods that are unfavorable for horizontal transmission or when there is a low mosquito density is not clearly understood, there are indications that vertical transmission is an important maintenance mechanism for DENV circulation during these periods^15^. Indeed, vertical transmission of this virus was observed in seven and three consecutive generations of *Ae. aegypti* and *Ae. albopictus*, respectively, under laboratory conditions^16,17^, confirming the importance of this phenomenon.

A recent review^15^ showed that Asia and South America are the main continents where vertical transmission of DENV in *Ae. aegypti* and *Ae. albopictus* under natural conditions has been reported. While Brazil is the leading country in the Americas on studies of natural vertical transmission of DENV, few studies have investigated this phenomenon in the state of São Paulo^18,19^.

The state of São Paulo is the most populous in Brazil, with approximately 45 million inhabitants, representing about 21.5% of the entire Brazilian population (209 million inhabitants)^20,21^. Despite the fact that São Paulo, the capital of the state, is the most populous city in Brazil and Latin America^22^ and in recent years has experienced major dengue epidemics, such as those in 2014 and 2015^23^, natural vertical transmission has never been detected in the city^15^.

The green areas in municipal parks in the city of São Paulo are remnants of Atlantic Forest vegetation. These parks are used for recreation and provide habitats for several species of mosquitoes of medical importance, such as those of the *Culex*, *Aedes* and *Anopheles* genera^24^. Previous studies performed in Piqueri Municipal Park, for example, reported a great abundance of *Ae. albopictus*^24,25^, followed by *Culex quinquefasciatus*, *Aedes fluviatilis*, *Aedes scapularis*, *Culex nigripalpu*s and *Ae. aegypti*^25^, corroborating the findings of studies that showed that *Ae. albopictus* is usually present in areas with vegetation cover^7,26^. Despite the body of knowledge on mosquito fauna in green areas in São Paulo, little is known about the pathogens circulating in these locations. Investigation into the occurrence of natural vertical transmission of DENV in *Ae. aegypti* and *Ae. albopictus* populations is therefore of critical importance for an understanding of the transmission dynamics of this virus and can provide a new outlook on the maintenance of this pathogen in urban green areas and its possible implications for public health.

This study therefore sought to investigate the occurrence of natural vertical transmission of DENV in *Ae. aegypti* and *Ae. albopictus* mosquito populations in the city of São Paulo.

## Results

### Mosquitoes

**Figure 1:**
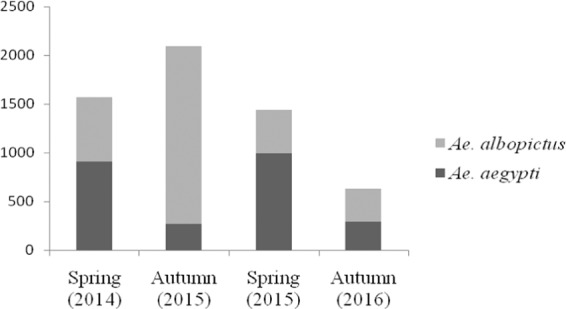
Number of mosquitoes analyzed for each species and season. Number of *Ae. aegypti* and *Ae. albopictus* specimens collected in Piqueri Municipal Park during two springs and two autumns between 2014 and 2016.

To detect natural vertical transmission of DENV in *Ae. aegypti* and *Ae. albopictus* during two seasons (spring and autumn), a total of 5,730 mosquito specimens were analyzed. Of these, 3,270 were *Ae. albopictus* (1,790 males and 1,480 females) and 2,460 *Ae. aegypti* (940 males and 1,520 females) (Table 1). Of the 5,730 specimens, 1,570 were collected in spring 2014, 2,090 in autumn 2015, 1,440 in spring 2015 and 630 in autumn 2016 (Fig. 1.)

**Table 1:** Mosquitoes analyzed.

	2014		2015				2016		
	Spring		Autumn		Spring		Autumn		
	Male	Female	Male	Female	Male	Female	Male	Female	**Total**
*Ae. aegypti*	210	700	90	180	580	410	60	230	**2,460**
*Ae. albopictus*	190	470	810	1,010	450	—	340	—	**3,270**
Total	**400**	**1,170**	**900**	**1,190**	**1,030**	**410**	**400**	**230**	**5,730**

Total number of mosquitoes analyzed separated by species, sex and season.

### Detection of positive pools

**Figure 2:**
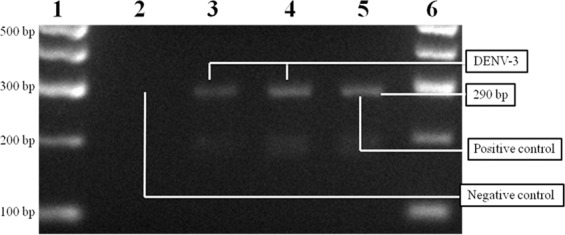
Electrophoresis in agarose gel of the RT-PCR products. Lanes 1 and 6: molecular weight marker; lane 2: PCR negative control; lane 3: DENV-3 genomic fragment from a pool of male *Ae. albopictus* collected during spring 2014; lane 4: DENV-3 genomic fragment from a pool of male *Ae. albopictus* collected during autumn 2015; lane 5: 290 bp amplified fragment of DENV-3 used as a positive control.

Of all the 573 pools tested, two of male *Ae. albopictus* were positive for DENV-3, showing a 290 bp band on agarose gel. One of the pools of male *Ae. albopictus* was collected in spring 2014 and the other in autumn 2015 (Fig. 2; Supplementary Figs. S1 and S2).

**Figure 3:**
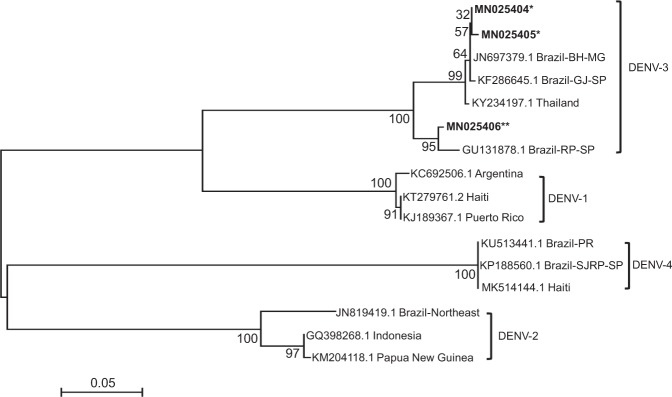
Neighbor Joining tree of DENV-3 C-PrM. Similarity tree of dengue virus serotypes sequences determined by MEGA software. Bootstrap values are shown on branch nodes. The two positive pools for DENV-3 sequences are shown (indicated by *) as well as the positive control (indicated by **).

The two positive samples were sequenced, edited, aligned with each other and compared with GenBank sequences using BLAST. They had high degree of identity with DENV-3 serotype sequences from GenBank, as shown in the Neighbor Joining tree (Fig. 3), and only three differences in nucleotide sequence were found between the two samples. Both showed high identity with strains found in the city of Guarujá, SP, Brazil, and Belo Horizonte, MG, Brazil. The positive control sample, which was also sequenced and aligned with GenBank sequences, showed high identity with strains of completely different origin. The DENV-3 sequences found in this study and the positive control sequence were deposited in GenBank, respectively, under accession numbers (MN025404/MN025405/MN025406), as well as the data of the sequences used for construction of the Neighbor Joining tree are described in Supplementary Table S1.

### Minimum infection rate (MIR)

We compared the overall Minimum Infection Rate (MIR = 0.35), which was calculated based on all the seasons and specimens studied, with the MIR for *Ae. albopictus* only (MIR = 0.61) and the MIR for *Ae. albopictus* only from each season that had positive pools (MIR spring 2014 = 1.47; MIR autumn 2015 = 0.44). The MIR for spring 2014 was higher than for autumn 2015.

## Discussion

Since the first reports of vertical transmission under natural conditions, the role of this phenomenon in the persistence and maintenance of arboviruses in nature has been the subject of much discussion^27,28^. The first report of natural vertical transmission of DENV in the Americas was described in specimens from Trinidad and Tobago^29^ and was subsequently reported in South American countries, including Bolivia^30^, Argentina^31^ and Peru^32^. In Brazil, the first report of natural vertical transmission was of DENV-1 in *Ae. albopictus* from the state of Minas Gerais^33^. Subsequently, other occurrences in this species involving other dengue serotypes were also reported in Brazil^18,34–36^. Natural vertical transmission of DENV in *Ae. aegypti* has been demonstrated in the states of Minas Gerais^35,37–39^, Pernambuco^40^, Ceará^36^, Mato Grosso^40–42^ Rio de Janeiro^43^ and Amazonas^44^.

Here, we report natural vertical transmission of DENV in *Ae. albopictus* for the first time in the city of São Paulo. Specifically, DENV-3 was detected in two pools of male *Ae. albopictus*. To date, only three studies have demonstrated natural vertical transmission of DENV-3 in Brazilian populations of *Ae. albopictus*, one in the state of Minas Gerais^35^, another in the state of Ceará^36^ and another in Santos (a coastal city in the state of São Paulo)^18^. Despite both DENV-3 positive samples of males of *Ae. albopictus* showed differences in nucleotide sequences, they exhibited high identity with strains found in the city of Guarujá, SP, Brazil, and Belo Horizonte, MG, Brazil, whereas the sequence of the positive control showed similarity with strains of the city of Ribeirão Preto, SP. The differences found between the three sequences indicate that the positive samples were not products of laboratory contamination.

Under laboratory conditions, females of *Ae. albopictus* were able to transmit all four DENV serotypes vertically to the offspring. The transmission rates varied according to the virus serotype and strain, and the lowest rate found was for DENV-3^45^. Although the maintenance of DENV-1 was observed in three consecutive generations of *Ae. albopictus* by vertical transmission^17^, there is no data about the DENV-3 maintenance over successive generations in this species under laboratory conditions. However, the persistence of DENV-3 was demonstrated in *Ae. aegypti* during seven successive generations^16^.

Vertical transmission was also observed in *Ae. aegypti* although at a low rate, even for strains known to be more susceptible to oral infection^45^. Similar results were found for a Brazilian DENV strain and populations of *Ae. aegypti* and *Ae. albopictus*^46^, indicating that despite the importance of *Ae. aegypti* for horizontal transmission this species may be less relevant in the maintenance of DENV during periods of low vector density^45,47^. Our results corroborate these findings as we found positive *Ae. albopictus* pools but no positive *Ae. aegypti* pools. Other hypothesis for the absence of positive pools of *Ae. aegypti* in our study is related to the smaller number of specimens analyzed compared to *Ae. albopictus*. In addition, green areas favor the presence and abundance of *Ae. albopictus* when compared to *Ae. aegypti*^24,25,48^ which maintains DENV circulation in areas with high concentrations of human beings and houses^49,50^. This may have increased the chances of finding viral circulation in *Ae. albopictus* and not in *Ae. aegypti*. The presence of DENV-3 in *Ae. albopictus* from Piqueri Municipal Park suggests that this mosquito may be acting in the transmission dynamics of DENV, thereby maintaining circulation of the arbovirus in this environment.

In a study conducted with the DENV-2 serotype and strains of *Ae. aegypti* mosquitoes with high and low susceptibility to infection, Mourya *et al*.^51^ argue that vertical transmission is more frequent in eggs allowed to hatch after longer periods, probably because of an increase in viral copy numbers in the eggs. It seems, therefore, that unfavorable environmental conditions, such as low availability of water, and the consequent increase in the time required for the eggs to hatch, favor vertical transmission. This may explain our findings of DENV-3 in *Ae. albopictus* during spring 2014 and autumn 2015, when there was lower rainfall (spring 2014: 5.1 mm; autumn 2015: 3.3 mm) than in the other periods (spring 2015: 26.5 mm; autumn 2016: 15.0 mm)^48^. Another possible explanation for our findings is that during these two periods more specimens were tested.

Interestingly, no autochthonous human cases of DENV-3 have been reported in the city of São Paulo since 2010^23^ (Supplementary Fig. S3). In fact, DENV-3 circulation was detected in the city of São Paulo in 2010^52^, but this serotype was not identified in this area during the epidemic of 2014^53^. This strengthens the hypothesis that vertical transmission could help maintain viral circulation during interepidemic periods and suggests that there is silent circulation of DENV-3 in green areas of São Paulo, a city with no detected autochthonous cases of infection by this serotype.

It is important to highlight that for natural vertical transmission to be detected under the conditions established in this study, an infected female had to have laid eggs in the ovitrap and successfully transmitted the virus vertically and the larvae had to have survived until adulthood to finally be analyzed. When mosquito infection rates are low, more specimens are required for a higher chance of virus detection. For example, for an infection rate of 1, a minimum of 1,609 and 2,301 mosquitoes are needed for an 80% and 90% chance of virus detection, respectively^54^. Although in our study we analyzed 2,460 *Ae. aegypti* adults and 3,270 *Ae. albopictus* adults that were raised in the laboratory from eggs collected in the park, if we consider that both species lay their eggs in more than one container in the same gonotrophic cycle^55,56^, it is possible that most of the mosquitoes analyzed originated from the same mother. Nevertheless, considering the difficulty in detecting this phenomenon in natural environments, the successful detection of natural vertical transmission of a serotype with no recent autochthonous cases in São Paulo^23^ in *Ae. albopictus* males in two different seasons indicates that the virus is circulating at significant rates in this vector population and being transmitted vertically.

The MIR values found in the present study are similar to those previously described in the literature, which are mainly low and vary considerably according to the mosquito population^57^. In addition, the fact that the MIR typically exhibits considerable temporal variability in vector populations^58^ explains the different results for the various seasons analyzed in this study.

As urban parks are open to the public, infected visitors can contribute to the expansion of several arboviruses. Furthermore, the continuous contact between humans, competent vectors and viruses make these parks high-risk areas for infection by DENV and other arboviruses, raising the question of the relevance of urban green areas in the transmission and maintenance dynamics of arboviruses^59–61^. Bearing in mind the biological characteristics of *Ae. albopictus*, such as its vector competence for dengue, yellow fever, Zika and chikungunya viruses in laboratory conditions^46,62–64^, our findings reinforce the importance of surveillance and control of this species as it may be playing a role in maintaining circulation of DENV, favoring infection of those who live close to parks or visitors to these parks and expanding the distribution of the virus to other areas.

## Methods

### Study area

**Figure 4:**
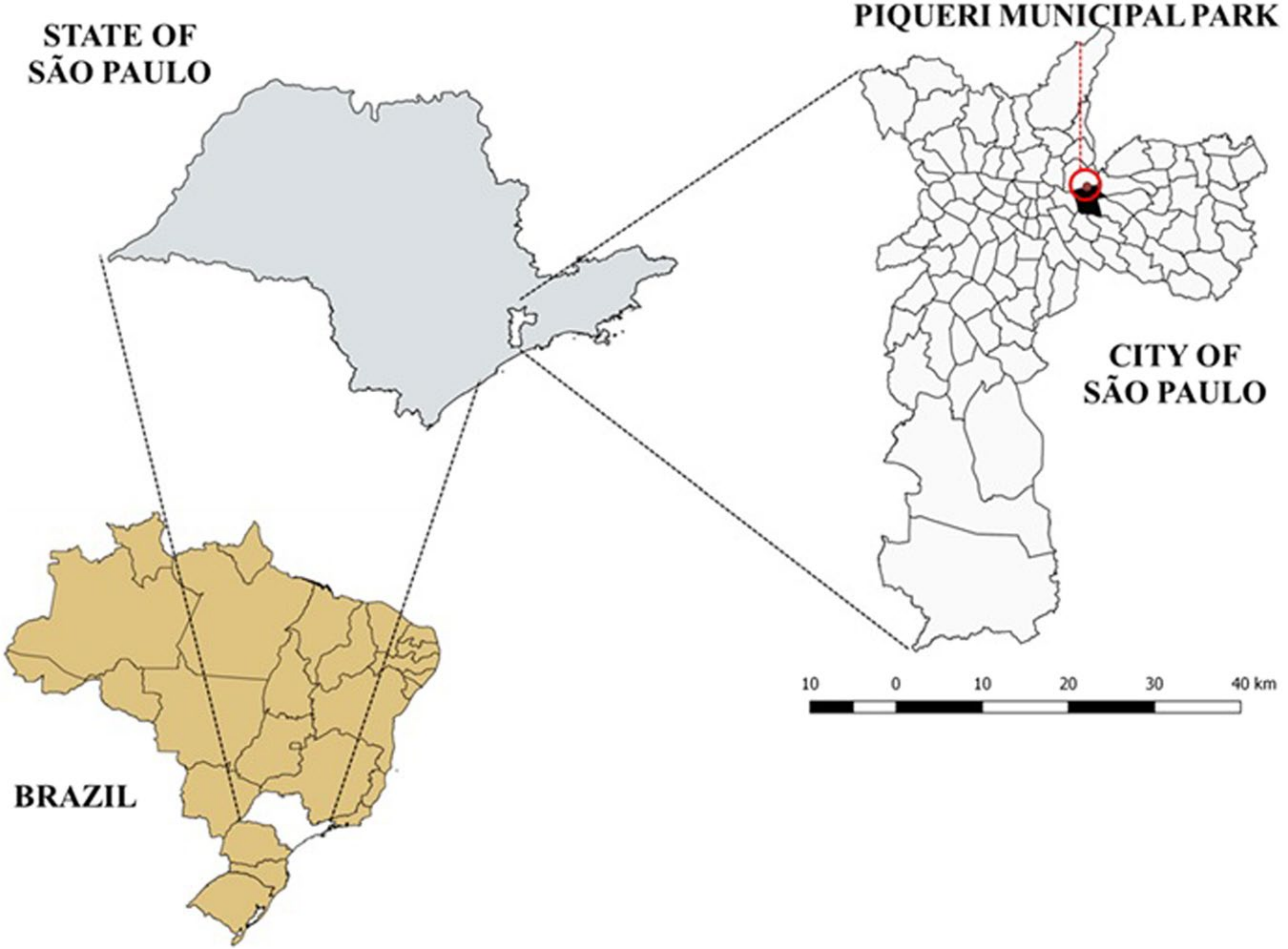
Study area. Location of Piqueri Municipal Park, Eastern São Paulo, SP.

To investigate the occurrence of vertical transmission of DENV in female and male *Ae. aegypti* and *Ae. albopictus* mosquitoes, adults were raised under laboratory conditions from eggs collected in urban green areas of Piqueri Municipal Park (23°31′39.98″S and 46°34′24.88″W), in Tatuapé district, in the east of the city of São Paulo, Brazil (Fig. 4). This park was selected as the study area because it is in the region of São Paulo that had one of the highest numbers of autochthonous human cases of dengue during the last major epidemic of the disease, in 2014/2015^23^. The park, which extends over approximately 100,000 m² and has dense vegetation, harbors approximately 90 species of animals^63^ and receives an estimated 36,000 visitors a month^63,64^.

### Entomological survey

Field collections were undertaken during six consecutive weeks in spring 2014 and spring 2015 and six consecutive weeks in autumn 2015 and autumn 2016. These seasons were chosen because of the moderate temperatures, which favor each species equally^48^.

Thirty-six ovitraps distributed around Piqueri Municipal Park were used to collect *Ae. aegypti* and *Ae. albopictus* eggs. All the paddles were collected and replaced weekly and taken to the Public Health Entomology Laboratory at the School of Public Health, University of São Paulo (FSP/USP).

### Mosquito rearing

The paddles were allowed to dry at room temperature for 3–4 days, assessed for the presence of eggs under a stereoscope and placed individually in plastic trays containing 500 mL of water and ground fish food (Tretramin®) for larval hatching. All larvae in the L4 stage were identified and separated by species (*Ae. aegypti* or *Ae. albopictus*). Pupae were placed individually in small glass containers, and after the adults emerged they were killed by freezing and separated by species, season and sex into pools of 10 individuals, which were placed in 1.5 mL polypropylene microtubes. All 573 pools were kept at −80 °C until used to isolate DENV.

### Isolation of viral genetic material

To detect the presence of DENV, each pool was initially macerated in 1 mL of Leibovitz’s L-15 medium and centrifuged for 15 minutes at 6000 rpm. A volume of 140 μL of the supernatant from each pool and the commercial QIAamp® Viral RNA Mini Kit (250) (QIAGEN, Hilden, Germany) were used for RNA extraction according to the manufacturer’s instructions. To avoid non-specific amplification, we treated the viral RNA with amplification-grade DNase I (Invitrogen, California, USA) following the manufacturer’s protocol.

RT-PCR was performed according to Lanciotti *et al*.^65^ with a second round of PCR amplification (semi-nested PCR) that allows simultaneous detection of the four DENV serotypes by generating DNA products of unique sizes, which can be used to differentiate the four serotypes (Table 2).

**Table 2:** Amplification of viral genetic material. Oligonucleotides used to amplify DENV serotypes.

Primer	Sequence (5′-3′)	Position in the genome	Amplified product size
D1	TCAATATGCTGAAACGCGCGAGAAACCG	134–161	511
D2	TTGCACCAACAGTCAATGTCTTCAGGTTC	616–644	511
TS1	CGTCTCAGTGATCCGGGGG	568–586	482 (D1 and TS1)
TS2	CGCCACAAGGGCCATGAACAG	232–252	119 (D1 and TS2)
TS3	TAACATCATCATGAGACAGAGC	400–421	290 (D1 and TS3)
TS4	CTCTGTTGTCTTAAACAAGAGA	506–527	392 (D1 and TS4)

The target sequence of the viral RNA was initially converted to cDNA by reverse transcriptase (RT) and then amplified using the consensus primers D1 and D2 and SuperScript® III One-Step RT-PCR with Platinum® Taq polymerase (Invitrogen, California, USA) according to the manufacturer’s protocol: 30 minutes at 50 °C and 2 minutes at 94 °C, followed by 35 cycles of denaturation (94 °C, 15 s), annealing (60 °C, 30 s) and extension (72 °C, 30 s). Semi-nested PCR was then performed using GoTaq Green Master Mix 2 ×(Promega, USA) and the following cycling conditions: 25 cycles of denaturation (95 °C, 30 s), annealing (60 °C, 30 s) and extension (72 °C, 1 min). RNA from the four DENV serotypes and ultrapure water were used as positive and negative controls, respectively. Bands were visualized under UV light on a 2% agarose gel stained with 5 μL of Gel-Red (Biotium). For double confirmation, all samples showing positive bands were reanalyzed from the extracted RNA using only the oligonucleotides specific to the serotype detected.

### Nucleotide sequencing

The resulting PCR products were purified with Exosap-IT (GE) following the manufacturer’s instructions. The purified DNA was then sequenced by the Sanger method using the Big Dye Terminator Sequencing Kit (ABI) and DENV serotype specific primers (Table 2) in the ABI 3100 Automated DNA Sequencer (ABI). The double-stranded DNA fragments were sequenced in both directions to generate a consensus double-stranded sequence for each sample. Editing, analysis and alignment of the nucleotides were conducted using version 6.0.7 of Bioedit Sequence Alignment Editor. All nucleotide sequences were aligned using ClustalW^66^, and sequence polymorphisms within the amplified region were identified by comparing our sequences with a variety of sequences of the four dengue virus serotypes available from GenBank (Supplementary Table S1). A similarity tree was built (Fig. 3**)** with Neighbor Joining algorithm, based on a Kimura 2-parameter model, nucleotide distances, determined by MEGA software (Molecular Evolutionary Genetics Analysis, version 6.0), with 1,000 replications in the bootstrap test^67^.

### Statistical analysis

The proportion of infected specimens is commonly estimated from the MIR (number of infected pools/total number of specimens x 1000 mosquitoes tested) based on the assumption of only one infected mosquito per positive pool^13,68^. This ratio is reasonable for estimates based on low-occurrence data and small pools, as observed in this study of natural vertical transmission^13,68^.

For the purposes of comparison, we calculated a general MIR for both species considering all the seasons studied (number of infected pools/total number of specimens for all the seasons analyzed x 1000), a MIR for *Ae. albopictus* only (number of infected pools of *Ae. albopictus*/total number of specimens analyzed for all seasons x 1000) and a MIR for *Ae. albopictus* for each season with positive pools (number of infected pools of *Ae. albopictus*/total number of specimens analyzed for each season with positive pools x 1,000).

## Supplementary information

Supplementary Information.

## Data Availability

All data generated or analyzed during this study are included in the manuscript.
