# Supplementary material for: Silent circulation of dengue virus in Aedes albopictus (Diptera: Culicidae) resulting from natural vertical transmission
Source: Sci Rep. 2020 Mar 2;10:3855. doi: 10.1038/s41598-020-60870-1 (PMC7052239; doi:10.1038/s41598-020-60870-1)
Supplement: Supplementary file 1 — Supplementary Information. [file 41598_2020_60870_MOESM1_ESM.pdf]

Silent circulation of dengue virus in *Aedes albopictus* (Diptera: Culicidae) resulting from natural vertical transmission

Authors: Victor Henrique Ferreira-de-Lima[1/*], Pâmela dos Santos Andrade[2/*], Luciano Matsumiya Thomazelli[3], Mauro Toledo Marrelli[2], Paulo Roberto Urbinatti[2], Rosa Maria Marques de Sá Almeida[2], Tamara Nunes Lima-Camara[2/+]

Affiliations:

[1]Institute of Tropical Medicine, University of São Paulo. Av. Dr. Enéas de Carvalho Aguiar, 470 - Jardim América, São Paulo - SP, 05403-000. Brazil. E-mail: victorhenriquelima@usp.br

[2]Department of Epidemiology, School of Public Health, University of São Paulo. Av. Dr. Arnaldo, 715 - Cerqueira César, São Paulo - SP, 03178-200. Brazil. E-mail: limacamara@usp.br

[3] Laboratory of Clinical and Molecular Virology (LVCM), Institute of Biomedical Sciences II, University of São Paulo. Av. Prof. Lineu Prestes, 1374 - Butantã, São Paulo – SP, 05508-900. Brazil. E-mail: lucmt@usp.br

[+]Corresponding author: limacamara@usp.br

*Equal contribution

Supplementary Information

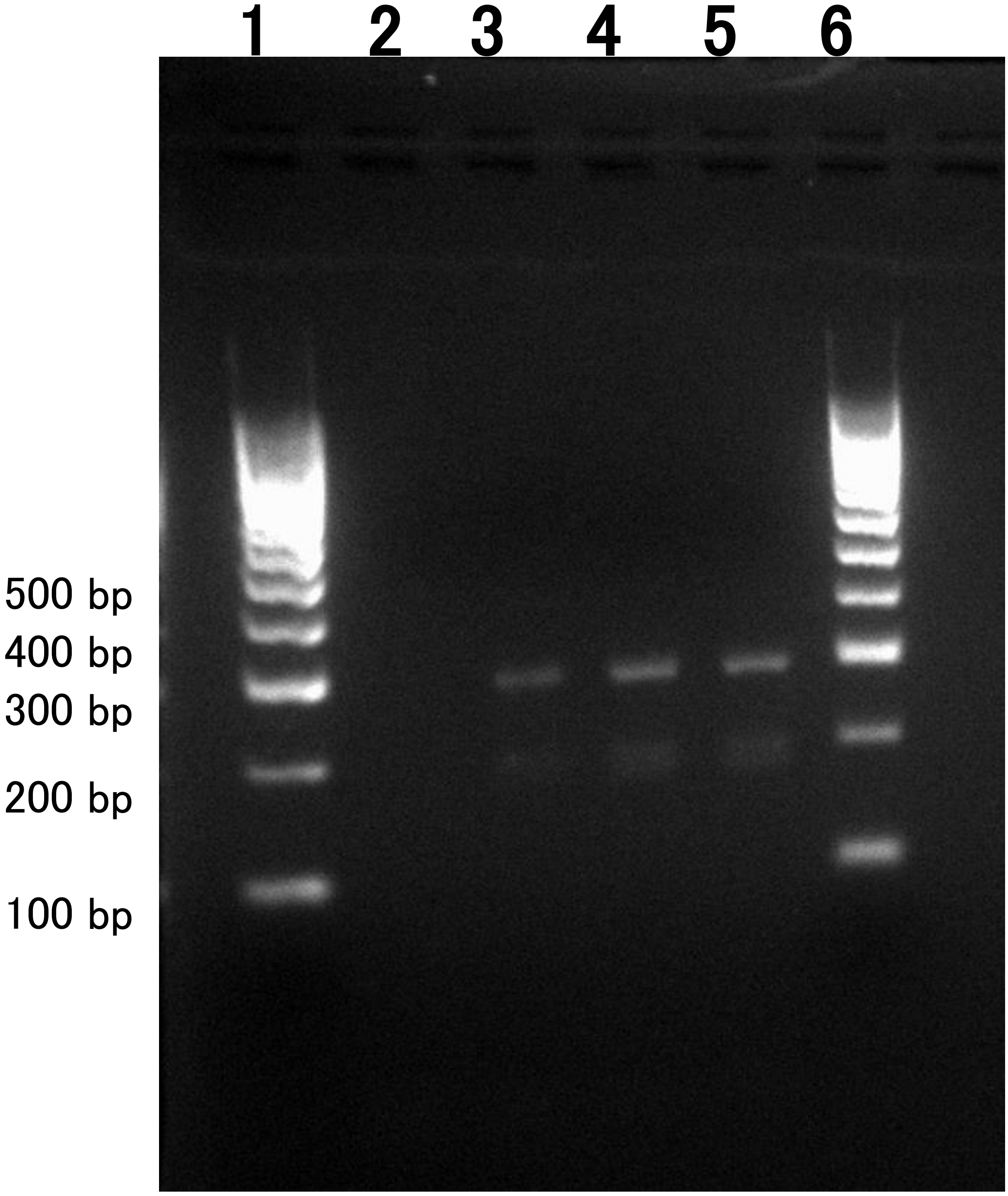

Fig S1. Full-length image of agarose gel of the RT-PCR products with contrast. Lanes 1 and 6: molecular weight marker; lane 2: PCR negative control; lane 3: DENV-3 genomic fragment from a pool of male *Ae. albopictus* collected during spring 2014; lane 4: DENV-3 genomic fragment from a pool of male *Ae. albopictus* collected during autumn 2015; lane 5: 290 bp amplified fragment of DENV-3 used as a positive control.

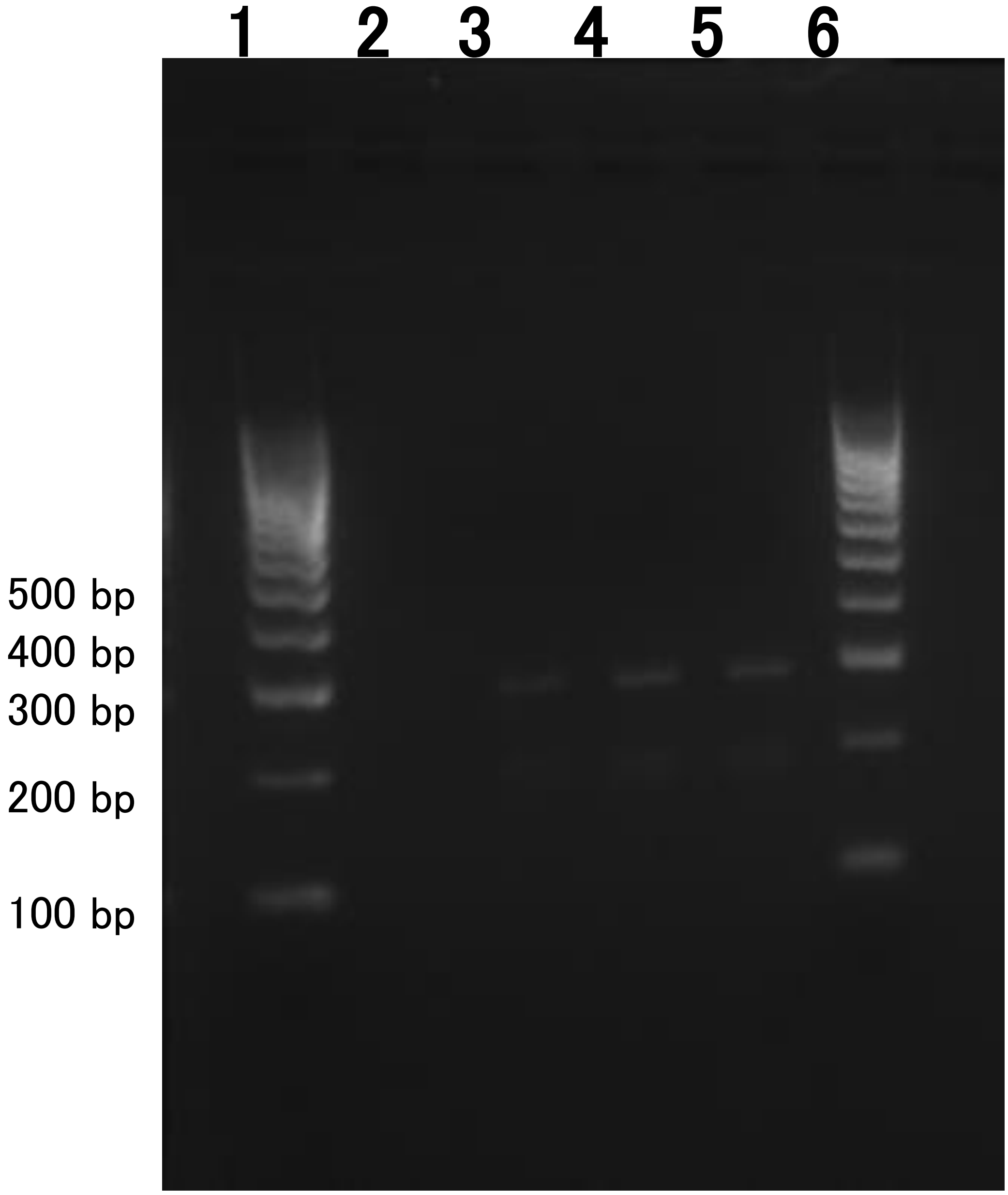

Fig S2. Full-length image of agarose gel of the RT-PCR products without contrast. Lanes 1 and 6: molecular weight marker; lane 2: PCR negative control; lane 3: DENV-3 genomic fragment from a pool of male *Ae. albopictus* collected during spring 2014; lane 4: DENV-3 genomic fragment from a pool of male *Ae. albopictus* collected during autumn 2015; lane 5: 290 bp amplified fragment of DENV-3 used as a positive control.

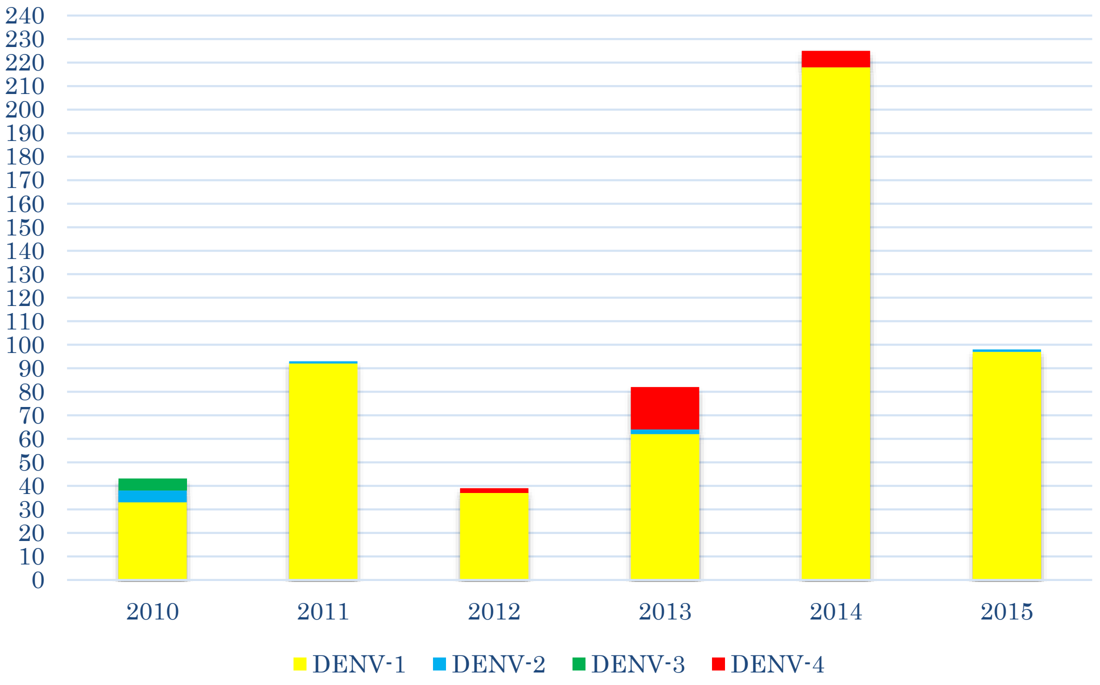

Fig S3. Dengue virus in the city of São Paulo. Dengue virus serotypes in autochthonous cases from the city of São Paulo, Brazil, 2010 to 2015[23].

Table S1. Similarity Tree. List of sequences of dengue virus serotypes from Genbank, according to geographic origin, serotype, year, host and accession number.

Geographic origin	Serotype	Year	Host	Genbank
Puerto Rico	DENV-1	2010	*Homo sapiens*	KJ189367.1
Haiti	DENV-1	2014	*Homo sapiens*	KT279761.2
Argentina	DENV-1	2009	*Homo sapiens*	KC692506.1
Brazil – Northeast Region	DENV-2	2000	*Homo sapiens*	JN819419.1
Papua New Guinea	DENV-2	1994	*Homo sapiens*	KM204118.1
Indonesia	DENV-2	1975	*Homo sapiens*	GQ398268.1
Thailand	DENV-3	2016	*Aedes aegypti*	KY234197.1
Brazil - Guaruja, SP	DENV-3	2013	*Homo sapiens*	KF286645.1
Brazil – Belo Horizonte, MG	DENV-3	2006	*Homo sapiens*	JN697379.1
Brazil – Ribeirão Preto SP	DENV-3	2007	*Homo sapiens*	GU131878.1
Brazil – São José do Rio Preto, SP	DENV-4	2012	*Homo sapiens*	KP188560.1
Haiti	DENV-4	2015	*Homo sapiens*	MK514144.1
Brazil – Cambe, PR	DENV-4	2013	*Homo sapiens*	KU513441.1
